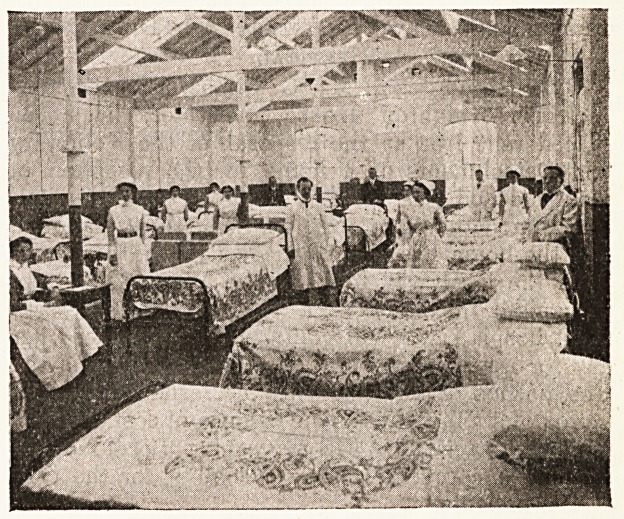# Hospitals and the War

**Published:** 1914-12-26

**Authors:** 


### HOSPITALS AND THE WAR.

### THE BOMBARDMENT OF THE HARTLEPOOLS: WORK AT THE CAMERON HOSPITAL.

Firing began at 8 a.m. on the 16th instant, and lasted forty minutes. The hospital suffered no damage whatever, and except for uneasiness and anxiety experienced by both patients and staff no ill effect was felt. Windows shook, and the noise from the bombardment was very severe.

During the bombardment motor-cars, cabs, etc., speedily conveyed the injured to the hospital. More than fifty serious cases were treated and detained, and 150 minor cases were also treated. Ten dead bodies were brought in, and nineteen patients have died from shock and wounds. There are now in hospital some twenty-five very serious cases. Most of these consist of mutilated limbs, requiring amputation. Over forty other serious cases were treated and had necessarily to be directed elsewhere.

### COVENTRY: WOUNDED IN THE NEW WARD.

The accompanying two illustrations give a good idea of the new ward at the Coventry and Warwickshire Hospital, which has been occupied of late by wounded soldiers. Known as the "Alfred Herbert" Ward, being given by the donor of that name who generously gave £1,000 for its establishment, it is proposed to take charge of 100 men until the end of the war. To meet the expense of maintenance, the chairman of the hospital committee, Mr. J. Wormell, asked for guarantees of the necessary funds to maintain one or more soldiers for specified periods, and his appeal has met with a numerous response.

The new ward itself, as its name implies, is really an addition to the institution, and formed originally a part of the Round Tower cycle factory in the grounds of the hospital. The task of adaptation was accomplished in ten days, and this has involved the laying down of a new floor, the insertion of new windows, and the installation of a kitchen and heating apparatus. As our two illustrations show, the factory may be contrasted with the ward into which it developed, and some idea of the work involved by it may be gauged from the fact that eight new nurses have had to be engaged for the administration and nursing. It was quickly occupied by arriving convoys, and by the beginning of this month it was three parts or more in occupation. The wounded have included both British and Belgian soldiers, and our illustrations preserve a record of a remarkable piece of emergency hospital work, of which so many instances have occurred throughout the country.

### LEICESTER: BASE HOSPITAL.

### Christmas Festivities.

A further convoy of 161 wounded and sick soldiers from the seat of war arrived in Leicester last week by the L. and N. W. Railway ambulance transport train from Southampton. The convoy was the sixteenth which had arrived since the beginning of the war. Of the present batch of patients thirty-nine were "cot" cases, and, as has recently been the case, the majority proved to be medical cases. The recent heavy rains in Flanders were responsible for many cases of rheumatism and allied conditions, and there were a certain number of frost-bite cases. Of the surgical cases twenty-six were slightly wounded and twelve severely. The transport to the 5th Northern General Hospital was again undertaken by Mr. A. W. Faire, the County Director Voluntary Aid Detachment.

On Thursday the hospital was visited by Sir Frederick Milner on behalf of the King, who spent several hours in conversing with every patient, and in handing to each, on His Majesty's behalf, a box of chocolates and a box of cigarettes, a gift much prized by the recipients.

Arrangements for the Christmas festivities have now been completed. Suitable gifts will be presented, as well as a huge Christmas cake, and an entertainment and conjuring exhibition have been arranged at the base hospital.

The military patients at the Leicester Royal Infirmary will be well looked after by Miss Vincent, the matron (who in her civil capacity and in her military capacity as principal matron of the base hospital will be busily employed), and each will receive, as well as every civil patient in the infirmary, a useful present on Christmas Day and share in the other festivities to be observed.

Freemasons' New Voluntary Aid Detachment Hospital.

By the kindness of Mr. G. Stibbe, a local manufacturer, Knighton House, Leicester, has been placed rent free at the disposal of the Voluntary Aid Detachments as a home for the reception of wounded and sick soldiers from the base hospital. Accommodation is provided for some thirty patients, and the home has been furnished by the Mayoress from the local war funds. The War Office have recognised the home as a Voluntary Aid Detachment hospital and will contribute 2s. per head per day for its maintenance. Over and above this the Freemasons of Leicester will be responsible for a period of six months for the balance of cost of maintenance, estimated at £18 per week. Local medical men have accepted the responsibility for the care of the patients.

### CAMBRIDGE: 1st EASTERN GENERAL HOSPITAL.

Royal Visits to the Wounded.

The British and Belgian wounded soldiers who are being cared for at the 1st Eastern General Hospital, Cambridge, were on Thursday last week visited by H.R.H. Princess Christian and H.H. Princess Victoria, who spent a considerable time conversing with many of the patients of the allied nations.

Analysis of Cases Admitted.

Colonel Griffiths furnished a tabulated analysis of the wounded admitted since the mobilisation on August 5, 1914. The first admission was on August 18, 1914, and the number of wounded admitted since is as follows:—

| Force | Officers | Men | Total | Deaths |
|---|---|---|---|---|
| Expeditionary ... | 6 | 2,134 | 2,140 | 7 = .32% |
| Belgians ... ... | — | 351 | 351 | 4 = 1.1% |
| Home Force ... | 4 | 874 | 878 | 6 = .68% |
| Totals ... | 10 | 3,359 | 3,369 | 17 = .51% |

There have been five cases of tetanus, two of which terminated fatally, twenty-two cases of pneumonia, with three deaths, and one case of a member of the Home Force dying from enteric fever.

### BRISTOL: RESULT OF CHOCOLATE SATURDAY.

On Saturday, December 12, Bristol received a contingent of about 152 patients who were suffering for the most part from the results of strain and shock; the larger proportion had been in hospital in France, although a few came direct from the Front. There were some twenty-eight "stretcher" cases.

Friday, December 18, saw the arrival of a further 200 sick and wounded, among them being men of the Grenadier Guards, King's Royal Rifles, Royal Engineers, and Yorkshire and Lancashire Regiments who had been in the base hospital at Boulogne.

The Bristol motor ambulance, which has been provided by public subscription, is now completed, and having undergone thorough and severe trials, is at the moment of writing awaiting orders for dispatch to the Front.

As the result of "Chocolate Saturday," which was held, it will be remembered, on December 5, between four and five tons of chocolate have been received by the Bristol Branch of the Navy League.

### CARDIFF: TERRITORIALS FROM THE TRENCHES.

On December 18, 140 more soldiers arrived at Cardiff, making 383 in eleven days. Happily there were not any "cot" cases, most of the men suffering from heart disease and rheumatism. There were only two or three Welshmen with the party, which had come direct from the trenches, having commenced the journey on the preceding Sunday.

One soldier said that after leaving Cardiff he went direct to the trenches, where he was for three weeks. The chief difficulty, he stated, was the rain, which was very heavy and incessant, and he had been sent home because he was "knocked up." He is the possessor of an interesting souvenir which he picked up on the battlefield: it is a pewter cup with the inscription "Souvenir de Londres," which he found near a church which the Germans had bombarded.

Among the men were some of the Liverpool Scottish (Territorials), who have been out since the beginning of November. One of them said that their clothing was only just "getting dry," as a result of being up to the waist in water. They expressed their delight at being back again, and added that they had received splendid treatment all along the journey.

### CHRISTMAS ARRANGEMENTS AT ST. BARTHOLOMEW'S HOSPITAL, ROCHESTER.

Many soldiers are being discharged on sick furlough this week in order that they may be able to spend Christmas in their own homes. Those remaining in the hospitals in the district will have as bright and comfortable a time as is possible under the circumstances. Already concerts and teas have been organised in the district for those who are able to take part in them, and it is anticipated that there will be numerous charitable gifts for those enforced to remain in hospital during the Christmas-tide; the Belgian soldiers will be particularly well looked after.

### TUNBRIDGE WELLS: VISITS FROM SOLDIERS' FRIENDS.

The number of wounded coming into the town has somewhat lessened lately, and the General Hospital has usually had beds vacant. Fourteen men were admitted early in the month, some with very nasty wounds, though not of a dangerous character, whilst others were for operation, and four were frost-bitten. Two men of the new Army were taken in for operation.

On Saturday, the 12th, the Right Hon. Sir Frederick Milner, Bart., visited the hospital at the request of the King and Queen to offer their Majesties' sympathy with the wounded men. Each man was spoken to separately and given a gift, a metal box filled with sweets, the box being adapted for use as a cigarette-case when emptied.

Every effort is being made to render the Christmas spent in the wards of the Tunbridge Wells General Hospital one which will not easily be forgotten. The soldiers will be able to have their friends with them on Christmas Day, and arrangements are being made to put up those who have to come from a distance.

**[Owing to pressure on our space accounts from some centres are unavoidably held over.]**

## Figures and Tables

**Figure f1:**
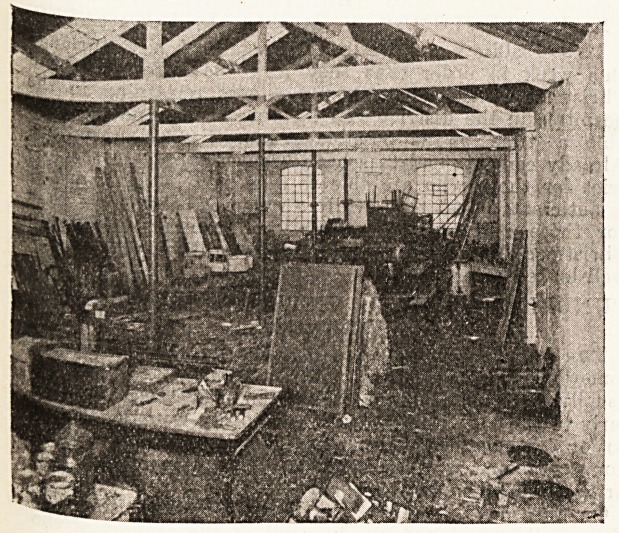


**Figure f2:**